# Prevalence of tick-borne haemoparasites in small ruminants in Turkey and diagnostic sensitivity of single-PCR and RLB

**DOI:** 10.1186/s13071-017-2151-3

**Published:** 2017-04-27

**Authors:** Huseyin Bilgin Bilgic, Serkan Bakırcı, Onur Kose, Ahmet Hakan Unlu, Selin Hacılarlıoglu, Hasan Eren, William Weir, Tulin Karagenc

**Affiliations:** 1 Department of Parasitology, University of Adnan Menderes, Faculty of Veterinary Medicine, 09016 Isıklı/Aydın, Turkey; 2 Department of Veterinary Medicine, University of Yuzuncu Yil, Vocational high School of Gevas, Programme of Laboratorian and Veterinary Health, 65700 Van, Turkey; 3 School of Veterinary Medicine, College of Medical, Veterinary and Life Sciences, University of Glasgow, Bearsden Road, Glasgow, G61 1QH UK

**Keywords:** Prevalence, Tick-borne pathogens, Sheep, Goat, RLB, Species-specific PCR, Turkey

## Abstract

**Background:**

Tick-borne haemoparasitic diseases (TBHDs), caused by *Theileria*, *Babesia*, *Anaplasma* and *Ehrlichia*, are common in regions of the world where the distributions of host, pathogen and vector overlap. Many of these diseases threaten livestock production and some also represent a concern to human public health. The primary aim of this study was to determine the prevalence of the above-mentioned pathogens in a large number of blood samples (*n* = 1979) collected from sheep (*n* = 1727) and goats (*n* = 252) in Turkey. A secondary aim was to assess the diagnostic sensitivity of a number of species-specific polymerase chain reaction (PCR) tests and the reverse line blotting (RLB) assay. DNA samples were screened using species-specific PCR for the presence of *Theileria ovis*, *Theileria* sp. MK, *T. lestoquardi*, *T. uilenbergi*, *T. luwenshuni*, *Babesia ovis*, *Anaplasma ovis* and *A. phagocytophilum* while RLB was undertaken to test for the presence of all known *Theileria*, *Babesia*, *Anaplasma* and *Ehrlichia* species. The diagnostic sensitivity of these two approaches was then compared in terms of their ability to detect single species and mixed infections.

**Results:**

Overall, 84 and 74.43% of the small ruminants sampled were identified as hosting one or more pathogen(s) by species-specific PCR and RLB respectively. The presence of *Theileria* sp. OT1, *T. luwenshuni* and *T. uilenbergi* in Turkey was revealed for the first time while the presence of *Babesia motasi*, *B. crassa* and *T. separata* in Turkish small ruminants was confirmed using molecular methods. A high prevalence of mixed infection was evident, with PCR and RLB approaches indicating that 52.24 and 35.42% of animals were co-infected with multiple species, respectively. More than 80% of the mixed infections contained *T. ovis* and/or *A. ovis*. The RLB approach was found to be capable of detecting mixed infections with species such as *Theileria* sp. OT1, *Theileria* sp. OT3, *T. separata*, *B. crassa* and *Babesia* spp.

**Conclusion:**

The results indicated that pathogens causing TBHDs are highly prevalent in sheep and goats in Turkey. The diagnostic sensitivity of species-specific single PCR was generally higher than that of RLB. However, the latter approach was still capable of identifying a high proportion of individuals containing mixed-species infections. The use of species-specific single PCR is recommended to accurately estimate pathogen prevalence and to identify co-infected hosts.

**Electronic supplementary material:**

The online version of this article (doi:10.1186/s13071-017-2151-3) contains supplementary material, which is available to authorized users.

## Background

Tick-borne haemoparasitic diseases (TBHDs) caused by protozoans (*Theileria*, *Babesia*) and bacteria (*Anaplasma/Ehrlichia*) impose a serious constraint upon livestock health and production in tropical and sub-tropical regions where the distributions of host, pathogen and vector overlap [1]. Over the past decade, much of the focus of TBHD research of livestock has been directed toward bovine pathogens and small ruminants have received limited consideration [2]. However, due to a growing appreciation of the socio-economic importance of small ruminants, more attention is now being directed toward pathogens of sheep and goats [2].

Haemoparasites observed in sheep and goats include *Theileria ovis*, *Theileria separata*, *Theileria* sp. OT1, *Theileria* sp. OT3, *Theileria* sp. MK, *Theileria lestoquardi* (formerly *T. hirci*), *Theileria luwenshuni* (*Theileria* sp. China 1), *Theileria uilenbergi* (*Theileria* sp. China 2), *Babesia ovis*, *Babesia motasi*, *Babesia crassa* [3–7] and *Anaplasma ovis*, *Anaplasma phagocytophilum*, *Ehrlichia ruminantium*, *Ehrlichia ovina* and *Ehrlichia* sp. Omatjenne [8–10]. *Babesia ovis*, *B. motasi*, *A. phagocytophilum*, *T. lestoquardi*, *T. luwenshuni* and *T. uilenbergi* are considered to be pathogenic, while *T. ovis*, *T. separata* and *B. crassa* are considered to be non-pathogenic in sheep and goats. No conclusive evidence yet exists about the pathogenicity of recently-described species such as *Theileria* sp. OT1, *Theileria* sp. OT3 and *Theileria* sp. MK [2, 5, 7, 9–14].

Losses attributed to TBHDs include mortality, production losses, veterinary diagnosis/treatment costs and tick control [15]. In small ruminants, TBHDs often manifest as sub-clinical infections. In animals that survive acute disease, a long-lasting carrier state develops, a condition that is associated with significant production and economic losses in the longer term [16, 17]. Additionally, carrier animals are important for the transmission of pathogens to feeding tick larvae, nymphs and/or adults. Over the last decade, molecular techniques such as species-specific polymerase chain reaction (PCR) and the reverse line blot (RLB) hybridisation assay have been widely used for detection and discrimination of *Theileria*, *Babesia* and *Anaplasma* species in animals [4, 8, 11, 18–20].

A number of studies on theileriosis and babesiosis of small ruminants have been previously performed in Turkey [12, 20, 21] and *T. ovis*, *Theileria* sp. MK, *Theileria* sp. OT3, and *B. ovis* have been detected in some parts of the country [12, 22]. However, *T. lestoquardi*, *T. luwenshuni*, *T. uilenbergi* and *Theileria* sp. OT1 have not been previously reported. *Anaplasma phagocytophilum* and *Ehrlichia* sp. Omatjenne have been identified in cattle and in ticks collected from animals and humans [23–26]. *Anaplasma ovis* was also detected in ticks collected from animal shelters [27], however only limited information exists concerning ovine anaplasmosis [28, 29].

In the present study, the prevalence of *Theileria*, *Babesia*, *Anaplasma* and *Ehrlichia* species in small ruminants in 18 different provinces of Turkey was determined using species-specific single PCR and RLB hybridisation. The diagnostic sensitivity of the two tests was then compared in terms of their ability to detect single and mixed infections.

## Methods

### Parasite material and sample collection

**Fig. 1:**
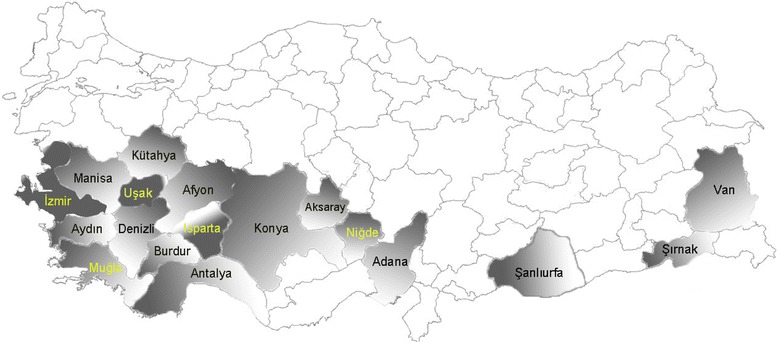
Map of Turkey showing the provinces of five geographical regions where the samples were collected

The study was conducted in 18 different provinces representing five different geographical regions of Turkey (Fig. 1). A total of 1979 blood samples were collected from sheep (*n* = 1727) and goats (*n* = 252) between 2011 and 2013 from the selected provinces. Blood samples were collected in EDTA tubes from randomly selected animals in each herd that were at least one year of age. DNA was extracted from blood samples using the Promega Wizard genomic DNA extraction kit (Madison, WI, USA) following the manufacturer’s instructions. Extracted DNA was resuspended in 100 μl elution buffer and stored at -20 °C until analysed. Control DNA samples used in this study included isolates of *T. ovis* (Kayseri), *Theileria* sp. MK (Kayseri) and *B. ovis* (Kayseri) from Turkey, isolates of *T. lestoquardi* (Lahr) and *B. crassa* from Iran, isolates of *T. uilenbergi* (Longde), *T. luwenshuni* (Lintan) and *B. motasi* (Lintan) from China and an isolate of *A. phagocytophilum* from the United Kingdom.

### RLB hybridisation assay for the detection of *Theileria*, *Babesia*, *Anaplasma* and *Ehrlichia* species

Samples were tested using the RLB hybridisation assay. The V4 hypervariable region of the 18S and V1 hypervariable region of the 16S small subunit ribosomal RNA gene of all *Theileria*, *Babesia*, *Ehrlichia* and *Anaplasma* species were amplified by PCR prior to RLB. Reactions were performed in a total volume of 50 μl containing 1× PCR buffer (ThermoFisher Scientific, Massachusetts, USA), 1.5 mM MgCl_2_ (Promega, Madison, WI, USA), 200 mM of each deoxynucleotide triphosphate (dNTP), 2.5 U of hot-start *Taq* polymerase (ThermoFisher Scientific, USA), 25 pmol of each primer: RLB-F, RLB-R (5′ biotin-labelled), Ehr-F and Ehr-R (5′-biotin labelled) (Additional file 1: Table S1) and 2 μl of template DNA. Reaction conditions comprised an initial denaturation step at 94 °C for 10 min followed by a touchdown programme employing two cycles at each temperature, i.e. 20 s at 94 °C, 30 s at an annealing temperature of 67 °C, 30 s at 72 °C, with the annealing temperature being decreased from 67 °C to 57 °C in steps of 2 °C. Following this, 40 cycles of 20 s at 94 °C, 30 s at 57 °C and 30 s at 72 °C were performed, followed by a final extension at 72 °C for 10 min. Oligonucleotide sequences used in this study are listed in Additional file 2: Table S2. 20 μl of each 18S and 16S biotin-labelled PCR products were screened by RLB hybridisation assay as previously described [4, 30] with a modification at the post-hybridisation washing step, which was performed at 52 °C for 10 min. After examination and documentation, the membrane was washed twice in preheated 1% SDS at 80 °C for 30 min, followed by washing in 100 ml 20 mM EDTA, pH 8, for 15 min at room temperature, then the membrane was covered in 20 mM EDTA, pH 8 and re-used about 15 times.

### Species-specific single PCR

All 1979 samples were screened with an array of species-specific single PCRs for the presence of *T. ovis*, *T. lestoquardi*, *T. uilenbergi*, *T. luwenshuni*, *Theileria* sp. MK, *B. ovis*, *A. ovis* and *A. phagocytophilum*. Details of primer pairs for each species are given in Additional file 1: Table S1. PCR reactions were performed in a final volume of 25 μl containing 10 mM Tris–HCl (pH 8.3), 50 mM KCl, 1.5 mM MgCl_2_, 0.001% gelatin, 250 μM of each deoxynucleotide triphosphate, 1 U of HOT FIREPol DNA polymerase (Solis Biodyne, Tartu, Estonia), 10 μM of forward and reverse primer and 2 μl of template DNA. The reactions were performed using an automatic thermal cycler (Techne, TC-512, Staffordshire, UK) and reaction conditions are given in Additional file 3: Table S3. For each reaction, 10 μl of PCR product were electrophoresed on a 1.5% agarose gel containing 10 μl/ml SybrGreen (SafeView™, ABM Inc., Richmond, Canada) in Tris-acetate-EDTA (TAE) buffer at 100 V and visualised under UV light.

### Cloning and sequencing

In order to confirm the specificity of the single PCRs, amplicons generated using *T. ovis*, *T. uilenbergi*, *T. luwenshuni*, *Theileria* sp. MK, *B. ovis*, *A. ovis* and *A.phagocytophilum* species-specific primers were sequenced. Briefly, gel purified (Qiagen Gel Purification Kit, Hilden, Germany) PCR products corresponding to each positive field sample, were cloned using the TOPO® TA Cloning® Kit (Invitrogen™, ThermoFisher Scientific, USA). Plasmids containing amplified PCR products were purified using a plasmid purification kit (Qiagen, Germany), then sequenced using a commercial service (GATC Biotech, Konstanz, Germany). *Theileria lestoquardi* was not identified in any of the samples collected for this study and therefore no sequences of this parasite could be generated.

### Statistical analysis

The Chi-square test was used to compare proportions of observed positivity in different regions and among different provinces. Observed differences were considered to be statistically significant when the resulting *P*-value was lower than 0.05. Agreement between the different diagnostic tests (PCR and RLB) assessing the presence of *A. ovis*, *T. ovis*, *T. luwenshuni*, *T. uilenbergi*, *Theileria* sp. MK and *B. ovis* was calculated. A kappa (κ) measure of agreement test was performed to compare the performance of the two tests; κ < 0 indicates no agreement beyond chance, while a κ-value between 0.81 and 0.99 indicates almost perfect agreement. A κ-value between 0.41 and 0.60 indicates a moderate level of agreement [31, 32].

## Results

### Prevalence of *Theileria*, *Babesia*, *Ehrlichia* and *Anaplasma* spp. determined by RLB

The prevalence and distribution of single and mixed-species infections are summarised in Table 1 and in Additional file 4: Table S4, respectively. The overall prevalence of haemoparasites detected by RLB was 74.78% with 1480 animals infected with at least one species. The prevalence of *Theileria* species was 64.1%, whereas for *Anaplasma* and *Babesia* species it was 41 and 9.9% respectively. The most prevalent *Theileria* spp. was *T. ovis* with a prevalence of 60% (1188/1979), followed by *Theileria* sp. OT1 (2.6%), *T. uilenbergi* (0.5%), *Theileria* sp. MK (0.4%) and *Theileria* sp. OT3 (0.2%). *Anaplasma ovis* was the only *Anaplasma* species detected by RLB with an overall prevalence of 41.1% (813/1979). An unclassified species of *Babesia* (denoted *Babesia* spp.) was found to be the most abundant (5.4%) *Babesia* species. The prevalence of *Babesia* spp. was found to be significantly higher (*χ* ^2^ = 16.2, *df* = 3, *P =* 0.001) in the Mediterranean region (7.3%) than in other regions. The total prevalences of *B. crassa* and *B. ovis* were 4.0 and 0.4%, respectively. *Babesia motasi* was detected only in two samples (0.1%). *Theileria lestoquardi*, *A. phagocytophilum*, *E. ruminantium*, *E. ovina* and *Ehrlichia* spp. Omatjenne were not detected by RLB in any of the samples tested.

**Table 1:** Single species infections detected by RLB and species-specific single PCR

Province	No. of animals	Detected by RLB (sheep/goat)										Detected by PCR (sheep/goat)							
		*A.o*	*T.o*	*T.sp.OT1*	*T.sp.OT3*	*T/B catchall*	*T.sp*	*B.o*	*B.sp*	*Bc*	*Bm*	*A.o*	*B.o*	*T.o*	*T.l.*	*T.u.*	*T.le*	*T.MK*	*A.p*
Adana	95	1/11	18	1								12/12		16					
Afyon	100	1	52									1		9					
Aksaray	55	6	18							1		10		10					
Antalya	95	8	25									9		10		9			
Aydın	273	9	64									28/1	2	44	10/1	1^a^		1	
Burdur	137	16/10	37	1						2		13/9		18	1^a^				
Denizli	140	3	53									4		21					
Isparta	55		20									1		11					
İzmir	104	1	39/1		1							2		20				8/1	
Konya	75	3	23				1			2		3		8					
Kütahya	103	33/1	3						1			37/1	1	3					
Manisa	101	1/10	19									5/30	1^a^	10					
Muğla	114	43^a^		2^a^		1^a^				1^a^	2^a^	51^a^	1^a^						7^a^
Niğde	214	20	50	2				1	2	1		29	4	18	13				
Şırnak	98	4/33	15/2							1^a^		2/30	1^a^	13/3					
Şanlıurfa	100	1	52							3		1		41					
Uşak	88	2	27							2		4		14		1		1	
Van	32	6	5									1		1		1			
Total	1979	223	523	6	1	1	1	1	3	13	2	296	10	270	25	12		11	7
Percentage		11.3	26.4	0.3					0.2	0.7	0.1	15	0.5	13.6	1.3	0.6		0.6	0.4

*Abbreviations*: *A.o Anaplasma ovis*, *B.o Babesia ovis*, *T.o Theileria ovis*, *T/B all* samples that showed reactivity with *Theileria/Babesia* catchall probe and considered as T/B genus positive, *T. sp* samples that showed reactivity with *Theileria* all probe and considered as *Theileria* spp. positive, *B. sp* samples that showed reactivity with *Babesia* all probe and considered as *Babesia* spp positive, *BcG Babesia crassa*, *Bm Babesia motasie*, *T.OT1 Theileria* sp. OT1, *T.OT3 Theileria* sp. OT3, *T.l Theileria luwenshuni*, *T.u Theileria uilenbergi*, *T.le Theileria lestoquardi*, *T.MK Theileria* sp. MK, *A.p Anaplasma phagocytophilum*

^**a**^Number of positive samples collected from goats

RLB detected single infections in 774 (39.1%) of the 1979 animals (Table 1). Among these single infections, the most prevalent species was *T. ovis* (26.4%), followed by *A. ovis* (11.3%), *B. crassa* (0.7%), *Theileria* sp. OT1 (0.3%) and *B. motasi* (0.1%). *Theileria ovis* and *A. ovis* were detected in all regions. The proportion of mixed infections detected by RLB was 35.42% indicating that 701/1979 animals were co-infected with multiple species (Additional file 4: Table S4). A variety of combinations of species was detected and the RLB was able to identify mixed infections with up to five species (Additional file 4: Table S4). A majority of the mixed infections (80.74%, 566/701) contained both *T. ovis* and *A. ovis*. Mixed infections, which did not include either of these species, were detected in only 11 (1.56%) animals.

The highest prevalence of overall infections detected by RLB was in the Southeastern and Eastern Region (87.83%) while the lowest (70.77%) was in the Aegean Region. The prevalence (52.61%) of single infections detected in southeastern / eastern region was significantly higher than other regions (*χ* ^2^ = 42.6, *df* = 6, *P <* 0.0001) (Additional file 5: Figure S1a). The RLB approach did not detect any *Babesia* spp. in the southeastern/eastern region, while *B. crassa* was observed in all regions and *B. ovis* was detected in four provinces (Niğde, Kutahya, Izmir and Şırnak). The prevalences of *T. uilenbergi* and *B. crassa* in southeastern/eastern region were 2.2 and 10.4%, respectively. Compared to other regions, significantly higher numbers of samples were found to be positive for both *T. uilenbergi* (*χ* ^2^ = 15.0, *df* = 3, *P =* 0.002) and *B. crassa* (*χ* ^2^ = 32.7, *df* = 3, *P <* 0.0001) in the southeastern/eastern region.

The highest prevalence of total infections (97.14%) detected by RLB was in Denizli while the lowest (34.73%) was in Antalya. *Theileria luwenshuni* and *T. separata* were both detected in two samples collected from Niğde province. In Antalya and Muğla, animals were solely infected with a single parasite species. In Afyon, Izmir, Manisa, Muğla, Şırnak and Şanlıurfa the prevalence of single infections was higher than that of mixed infections (Additional file 5: Figure S1b).

### Prevalence of haemoparasite species determined by PCR

The 1979 samples were screened with species-specific single PCRs for the presence of *T. ovis*, *T. lestoquardi*, *T. uilenbergi*, *T. luwenshuni*, *Theileria* sp. MK, *B. ovis*, *A. ovis* and *A. phagocytophilum*. The prevalence and distribution of single and mixed infections detected by PCR are given in Table 1 and in Additional file 6: Table S5, respectively. An overall prevalence was determined to be 84.1%, indicating 1665/1979 animals were infected with at least one species. The prevalence of the two most abundant species, *A. ovis* (63.3%) and *T. ovis* (61.4%), were very similar and much higher than the other species. None of the collected samples were found to be positive for *T. lestoquardi* by PCR.

**Fig. 2:**
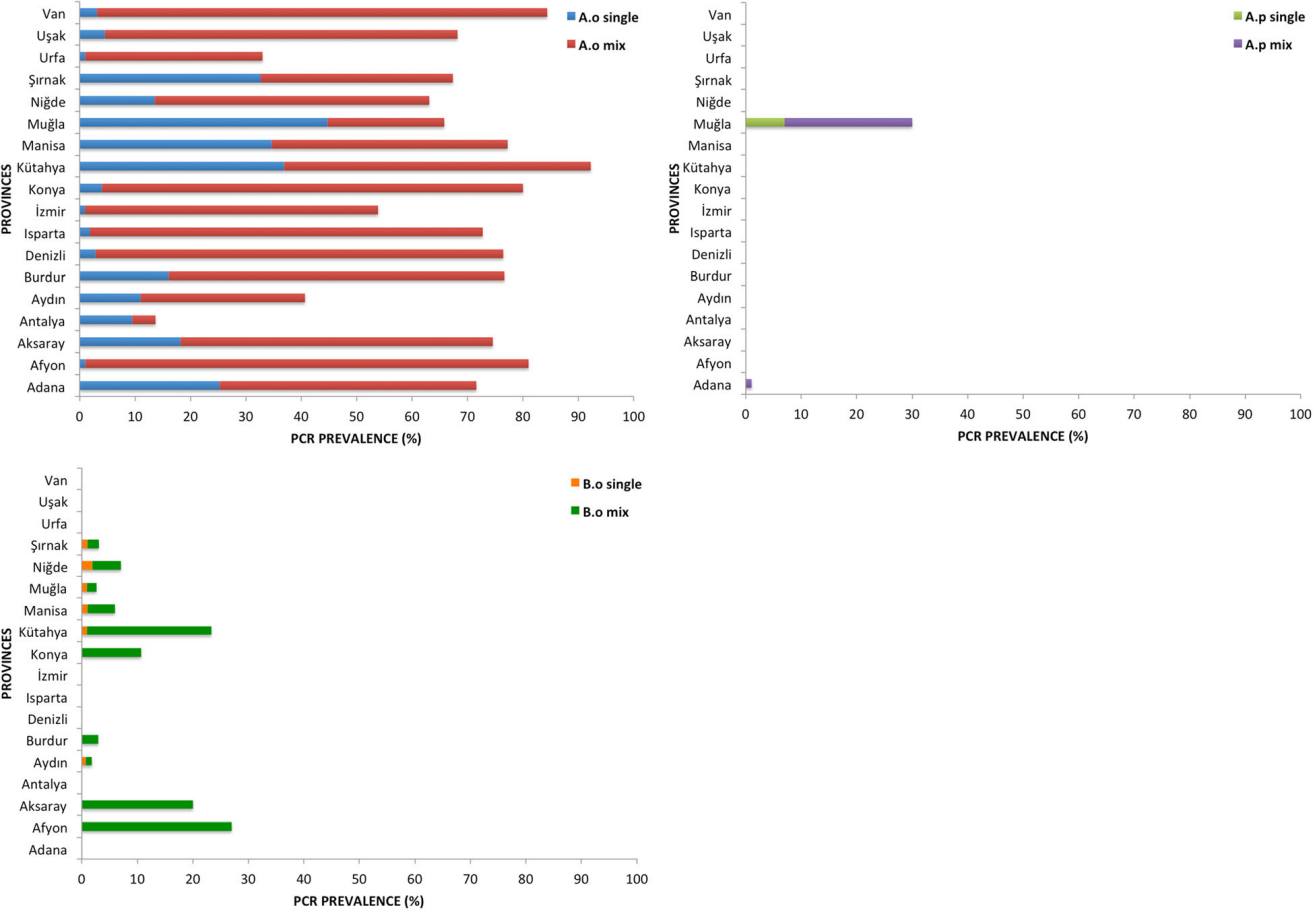
Distribution of infections detected by *A. ovis*, *A. phagocytophilum* and *B. ovis* PCRs among provinces. *Abbreviations*: *A.o*, *Anaplasma ovis*; *A.p*, *Anaplasma phagocytophilum; B.o*, *Babesia ovis*

The highest prevalence of *A. ovis* was found in the Central Anatolia region (68.7%) with a statistically significant difference (*χ* ^2^ = 15.1, *df* = 3, *P =* 0.002) among regions. *Anaplasma ovis* was detected in all provinces with the highest prevalence detected in Kütahya (92%) and the lowest in Antalya (13%) (Fig. 2). The prevalence of *A. ovis* also differed significantly (*χ* ^2^ = 308.9, *df* = 17, *P <* 0.0001) among provinces. The prevalence of *A. phagocytophilum* (2.9%) detected in the Aegean region was significantly higher than other regions (*χ* ^2^ = 25.7, *df* = 3, *P* < 0.0001). This pathogen was only detected in goats in Muğla and Adana provinces (Fig. 2).

**Fig. 3:**
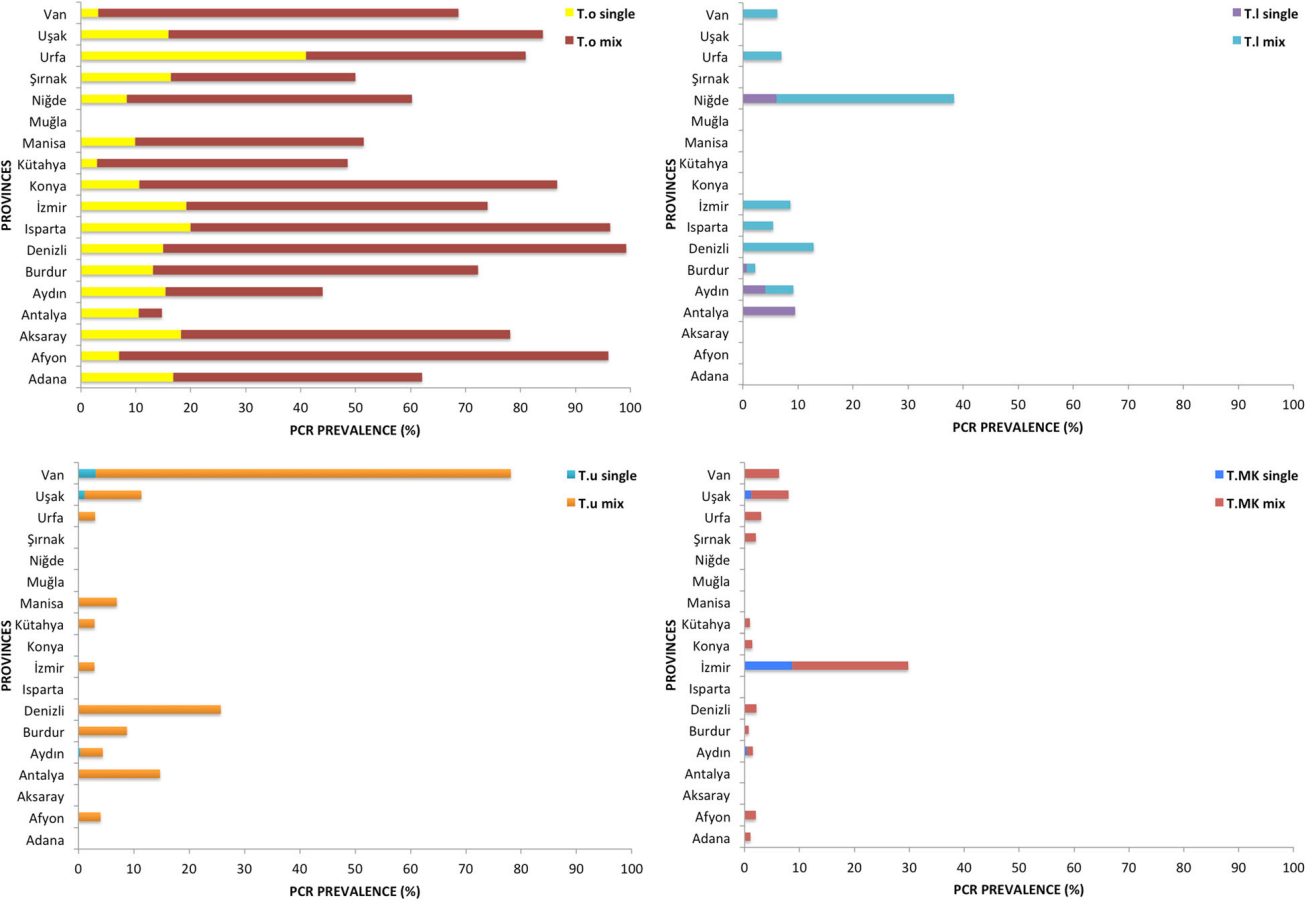
Distribution of infections detected by PCRs specific for *Theileria* species among provinces. *Abbreviations*: *T.o*, *Theileria ovis*; *T.l*, *Theileria luwenshuni*; *T.u*, *Theileria uilenbergi*; *T.le*, *Theileria lestoquardi* and *T.*MK *Theileria* sp. MK

*Theileria ovis* was detected in all provinces, except Muğla. The highest infection rate was found in Afyon (97%) and Isparta (96.4%) and the lowest was in Antalya (14.7%) (Fig. 3) and this difference was statistically significant (*χ* ^2^ = 537.6, *df* = 17, *P <* 0.0001). The prevalence of *T. ovis* was found to be significantly different among regions (*χ* ^2^ = 14.7, *df* = 3, *P =* 0.002) with the highest prevalences found in Central Anatolia (68.9%) and southeastern/eastern regions (66.1%). The prevalence of *T. luwenshuni* infection was statistically significantly different (*χ* ^2^ = 163,9, *df* = 3, *P <* 0.0001) among regions and the highest prevalence was found in the Central Anatolia region (23.8%). The highest infection rate was found in Niğde (38.3%) and lowest was in Burdur (2%) with a significance difference (*χ* ^2^ = 379.6, *df* = 17, *P <* 0.0001) among provinces.

The prevalence of *T. uilenbergi* was significantly different among regions (*χ* ^2^ = 35.6, *df* = 3, *P <* 0.0001) and the highest prevalence was in the southeastern/eastern region (11.7%). The highest infection rate was found in Van (75%), while the infection rate in Izmir (3%), Kütahya (3%) and Şanlıurfa (3%) provinces was much lower (Fig. 3). The difference among provinces was statistically significant (*χ* ^2^ = 407.7, *df* = 17, *P <* 0.0001). The prevalence of *Theileria* sp. MK was statistically significantly different among regions (*χ* ^2^ = 27.3, *df* = 3, *P <* 0.0001) with the highest prevalence detected in Aegean region (4.7%).

PCR detected a total of 106 infections with *B. ovis* both as a single (0.5%) and mixed infection (4.9%) with up to four species. The prevalence of *B. ovis* was found to be significantly different among regions (*χ* ^2^ = 37.3, *df* = 3, *P* < 0.0001) and the highest prevalence was found in Central Anatolia region (9.9%). The prevalence of *B. ovis* in Afyon (27%) was significantly higher than other provinces (*χ* ^2^ = 237.6, *df* = 17, *P <* 0.0001).

PCR detected single infections in 631 (32%) of 1979 animals, while mixed infections were observed in 52.2% of samples. Among samples with single infections, *A. ovis* and *T. ovis* were found at a considerably higher level than any other species. PCR results revealed that 28 different combinations of mixed infections occurred, some with up to four species (Additional file 6: Table S5). Of the 1034 samples containing a mixture of species, 64.3% (663/1031) contained both *T. ovis* and *A. ovis* and, overall, 99.61% of samples with mixed infections (1029/1031) had at least one of these species present. The prevalence of mixed infections detected by PCR was found to be statistically significantly different among regions (*χ* ^2^ = 38.09, *df* = 6, *P* < 0.0001) and the highest prevalence was found in the Aegean region (27.13%).

### Comparison of species-specific, single-PCR and RLB tests

The observed prevalences of *T. ovis* (*χ* ^2^ = 998.4, *df* = 1, *P <* 0.0001), *T. luwenshuni* (*χ* ^2^ = 12.2, *df* = 1, *P <* 0.0001), *Theileria* sp. MK (*χ* ^2^ = 169.2, *df* = 1, *P <* 0.0001) and *B. ovis* (*χ* ^2^ = 51.7, *df* = 1, *P <* 0.0001) by PCR were significantly higher than that of RLB. Additionally, PCR detected a significantly higher (*χ* ^2^ = 285.7, *df* = 1, *P <* 0.0001) number of *A. ovis* (1253/1979) infections compared to RLB. For *T. uilenbergi*, no significant difference was observed (Table 2). The kappa values when comparing PCR and RLB tests for *A. ovis* and *T. ovis* indicated a moderate (κ = 0.344) and a substantial (κ = 0.710) level of agreement, respectively, while for *T. luwenshuni*, *T. uilenbergi*, *Theileria* sp. MK and *B. ovis* the kappa values ranged from below zero to 0.179 indicating no agreement between PCR and RLB tests (Table 2).

**Table 2:** Comparison of PCR and RLB results

Species			PCR		Total	*P*-value	Measurement of agreement^a^	
			Positive	Negative			Kappa value	SD (95% CI)
*A. ovis*	RLB	Positive	693	120	813	< 0.0001*	0.344	0.019
		Negative	560	606	1166			
	Total		1253	726	1979			
*T. ovis*	RLB	Positive	1065	123	1188	< 0.0001*	0.710	0.016
		Negative	150	641	791			
	Total		1215	764	1979			
*T. luwenshuni*	RLB	Positive	1	0	1	< 0.0001*	0.012	0.012
		Negative	148	1830	1978			
	Total		149	1830	1979			
*T. uilenbergi*	RLB	Positive	0	10	10	0.404	-0.009	0.003
		Negative	128	1841	1969			
	Total		128	1851	1979			
*Theileria* sp. MK	RLB	Positive	6	1	7	< 0.0001*	0.179	0.064
		Negative	52	1920	1972			
	Total		58	1921	1979			
*B. ovis*	RLB	Positive	5	3	8	< 0.0001*	0.081	0.035
		Negative	101	1870	1971			
	Total		106	1873	1979			
*A. phagocytophilum*	RLB	Positive	0	0	0	–	–	–
		Negative	31	1948	1979			
	Total		31	1948	1979			

*P-values considered as statistically significant (*P* < 0.05) based on the Chi-square test

^a^Agreement expressed as kappa value when comparing PCR and RLB tests for *A. ovis*, *T. ovis*, *T. luwenshuni*, *T. uilenbergi*, *Theileria* sp. MK, *B. ovis* and *A. phagocytophilum* two-by-two. None of the collected samples were found to be positive for *T. lestoquardi* by PCR and RLB

Mixed infection was common among small ruminants and PCR results showed that 52.24% (1034/1979) of the samples were co-infected, while RLB detected a co-infection rate of 35.42% (701/1979). The observed prevalence of mixed infections detected by PCR was significantly higher than that of RLB (*χ* ^2^ = 586.7, *df* = 4, *P <* 0.0001, Table 3). The kappa value was 0.288 when comparing PCR and RLB tests for the presence of single and mixed infections indicating a slight or weak agreement between two tests [31, 32].

**Table 3:** Comparison of single and mixed species infections detected by RLB and species-specific single PCR

Type of infection		RLB			Total	*P*-value	Measurement of agreement	
		Single	Mixed	Negative			Kappa value	SD (95% CI)
PCR	Single	326	109	196	631			
	Mixed	391	536	107	1034			
	Negative	57	56	201	314			
	Total	774	701	504	1979	< 0.0001*	0.288	0.017

*P-values considered as statistically significant (*P* < 0.05) based on the Chi-square test

### Sequence analysis

The specificity of the single PCRs was confirmed by sequencing PCR amplicons generated using *T. ovis*, *T. uilenbergi*, *T. luwenshuni*, *Theileria* sp. MK, *B. ovis*, *A. ovis* and *A. phagocytophilum* species-specific primer sets. When the sequence of each amplified fragment was compared with reference sequences in the NCBI database, the 520 bp *T. ovis* product (GenBank KY283961), the 388 bp *T. luwenshuni* product (GenBank KY283963) and the 870 bp *A. ovis* product (GenBank KY283958) showed 100% identity with isolates and clones available in the database. The 389 bp *T. uilenbergi* product (GenBank KY283964) showed 100% identity with 18S ribosomal RNA genes of *T. uilenbergi* isolate Li 2 and the 757 bp *Theileria* sp. MK product (GenBank KY283962) showed 99% identity with 18S ribosomal RNA genes of *Theileria* sp. MK from sheep. The 549 bp *B. ovis* product (GenBank KY283960) showed 99% identity with small subunit ribosomal RNA genes of a number of *B. ovis* isolates. The 849 bp *A. phagocytophilum* product (GenBank KY283959) showed 97% identity to *msp* genes of a number of *A. phagocytophilum* isolates (data not shown).

## Discussion

### Prevalence of TBHDs detected by PCR and RLB

Despite the widespread distribution of ovine tick-borne haemoparasitic diseases in tropical and subtropical regions of the world [33] and their constraint to livestock production [1, 15, 29, 34], relatively limited information on their abundance and distribution is currently available [33]. Theileriosis and babesiosis are the two most extensively studied tick-borne haemoparasitic diseases occurring in small ruminants in Turkey; however only limited information exists on ovine anaplasmosis. In the present study, a comprehensive investigation of the distribution and prevalence of ovine haemoparasites in 18 provinces of Turkey was undertaken. To this end, two approaches were utilised, RLB hybridisation assay and species-specific PCR.

In previous studies, *Theileria* species such as *Theileria* sp. (15%), *T. ovis* (15–92%), *Theileria* sp. MK (1–2%) and *Theileria* sp. OT3 (0.4%) were detected in Central Anatolia [21, 35], Eastern and Southeastern Anatolia and the Black Sea region [12, 20] of Turkey. In this study, RLB (60%) and PCR (61.4%) results revealed that *T. ovis* is the most prevalent parasite in all provinces, except in Mugla. *Theileria ovis* is transmitted by *Rhipicephalus* ticks trans-stadially [36] and these ticks play an important role in the transmission of the parasite in Turkey [20]. *Theileria ovis* has also been detected in salivary glands of *R. bursa* [37] which was reported as the major species of tick found on sheep and goats in the Black Sea region [38]. Considering that evidence demonstrating the existence of *Rhipicephalus* species such as *R. bursa*, *R. sanguineus* and *R. turanicus* in Mugla (unpublished data), it is unclear why *T. ovis* was not be detected in the present study. In this province samples were mostly collected from domestic goats; whether widening the sampling to sheep would result in detection of the parasite in this region remains to be determined.

The results obtained from the RLB assay revealed a low prevalence of *Theileria* sp. MK (0.4%) and *Theileria* sp. OT3 (0.2%), similar to previously reported prevalence rates in East Anatolia and the Black Sea region [12, 20, 39]. A higher prevalence of *Theileria* sp. MK was noted (2.9%) when samples are screened by species-specific PCR. At present, no reliable information exists about the pathogenicity and vectors of these species in Turkey.

*Theileria luwenshuni* and *T. uilenbergi* are considered to be highly pathogenic for small ruminants [2]. These two parasites have been detected in China [3, 40] and Iraq [29] and recent evidence suggests the distribution of *T. luwenshuni* extends as far as the UK [41]. A study conducted in Iran revealed the presence of *T. luwenshuni* in sheepdog isolates [42]. The present study revealed the presence of *Theileria* sp. OT1, *T. luwenshuni* and *T. uilenbergi* in Turkey for the first time, demonstrating a broader distribution of these species than previously assumed. The distribution of *T. luwenshuni* and *T. uilenbergi* extended from the far east of the country (Van) to the far west (Izmir) of Turkey encompassing provinces located in different regions (Fig. 3). These species occurred as co-infections rather than single infections. *Theileria uilenbergi* has previously been detected as a co-infection of sheep with *A. ovis* and/or *T. ovis* in northern Iraq [29]. There is a possibility that *T. luwenshuni* and *T. uilenbergi* have been introduced to Turkey from northern Iraq and/or Iran with the movement/migratory pattern of domestic small ruminants and/or wild ruminants. Alternatively, they may have existed undetected in Turkey for a considerable period. *Haemaphysalis qinghaiensis* and *H. longicornis* have been shown to be responsible for transmitting *T. luwenshuni* and *T. uilenbergi* in China [43]. A relationship between *T. luwenshuni*/*Theileria* sp. OT1 and a *Haemaphysalis* spp. was suggested in a previous study [33] and *H. punctata* nymphs were suspected of the introduction of *T. luwenshuni* to the UK [41]. *Haemaphysalis* spp. such as *H. parva*, *H. sulcata* and *H. punctata* have been detected in various regions of Turkey [44]. *Theileria lestoquardi*, the cause of malignant theileriosis, has never been documented in Turkey, which is surprising since it has been detected in the neighbouring countries of Iran [45] and Iraq [29].

*Babesia ovis*, *B. motasi* and *B. crassa* cause ovine babesiosis in small ruminants [7]. Of these species, *B. ovis* is highly pathogenic for sheep and goats [46] and has been detected in Central, Eastern and South-eastern Anatolia regions [21, 22] with a prevalence ranging from 2 to 41%. Results gathered in this study indicated a broader distribution of *B. ovis* in Aegean and Mediterranean regions compared to other regions (Fig. 2). *Babesia motasi* and *B. crassa* are considered to be pathogenic and mild or non-pathogenic for sheep and goats, respectively [7]. *Haemaphysalis* ticks are responsible for transmitting *B. motasi* [7] and *B. crassa* has been detected in *Haemaphysalis* ticks in both continental Europe and in Turkey [47, 48]. *Theileria separata* has been described as a non-pathogenic parasite of sheep [49] and *Rhipicephalus evertsi* has been implicated in its transmission [50]. In Turkey, the presence of *B. motasi*, *B. crassa* and *T. separata* in small ruminants has been suspected for several years. Although, *Babesia motasi* infection in sheep and goats has been reported in Mediterranean, Central Anatolia and Eastern regions of Turkey between 1954 and 1989 [51], it was unknown whether this pathogen is currently present. *Babesia crassa* has been detected in ixodid ticks collected from humans [24] while *T. separata* has not been reported previously. This is the first molecular confirmation of *B. motasi*, *B. crassa* and *T. separata* in small ruminants in Turkey. Results indicate that the ovine *Babesia* species detected in this study were sporadically distributed in the Aegean, Mediterranean, eastern and southeastern Anatolia regions. The distribution of *Babesia* spp. overlaps with the distribution of transmitting *Rhipicephalus* and *Haemaphysalis* ticks [7]. In this study, an unclassified *Babesia* spp. was also detected by RLB with a prevalence of 5.4% and, like *B. crassa*, it was abundantly detected in concurrent infections with other species (5.25%).

Ovine anaplasmosis caused by *A. ovis*, is a tick-borne disease affecting sheep and goats [52]. Distribution of *A. ovis* extends from northern and southern Europe, Hungary to the Middle East and Cyprus [53–56]. In Turkey, *A. ovis* has been detected in ticks collected from animal shelters [27]. It has been proposed that relatively low productivity of local breeds might be related to infection with various tick-borne pathogens, including *A. ovis* [51]. *Anaplasma ovis* infection may predispose small ruminants to other fatal diseases and severe diseases have been described in bighorn sheep [57] and goats [58]. Of the tick-borne haemoparasitic diseases investigated in the present study, ovine anaplasmosis was found to be one of the most prevalent with approximately 70% of animals carrying *A. ovis*. Approximately 80% of the mixed infections detected in the present study were found to involve *A. ovis*, however, no clear impact of *A. ovis* infection in terms of clinical significance could be deduced. The existence of ixodid ticks transmitting *A. ovis* such as *Haemaphysalis*, *Dermacentor*, *Rhipicephalus* and *Hyalomma* [43, 59] has been reported in Central Anatolia region and in Aegean region [44] with their seasonal activity affecting the distribution of disease.

*Anaplasma phagocytophilum* is a pathogenic rickettsial organism causing tick-borne fever (TBF) in domestic animals and wild ruminants [9] with a world-wide distribution [60–62]. It was previously identified in domestic farm animals in Turkey [26, 28] although limited information on its impact on small ruminants exists [23, 28]. In the present study, *A. phagocytophilum* was only detected in goats, with 30 of the 31 positives coming from Mugla province. Both single (22.6%) and mixed (77.4%) infections were detected including one animal found to be co-infected with *A. ovis*. In previous studies, the prevalence of co-infections with *A. ovis* and *A. phagocytophilum* ranged from 6.5 to 22.22% in sheep with only a single infection with *A. ovis* detected in goats [55, 56]. *Ixodes ricinus* is the main vector of *A. phagocytophilum* throughout Europe. In Turkey, *A. phagocytophilum* has been detected in tick species like *Haemaphysalis sulcata*, *Rhipicephalus bursa* and *Ixodes ricinus* [23–25]. It is accepted that *A. phagocytophilum* is not host specific and that human isolates may cause infections in animals and vice versa [63]. Therefore, the higher prevalence of *A. phagocytophilum* detected in the present study suggests that the risk of transmitting this pathogen to humans may be higher than previously anticipated. It is important to note that *A. phagocytophilum* was detected in wild goats inhabiting the Marmaris National Park within the Mugla Province (unpublished data). As there is no physical barrier between wildlife and grazing animals in this area, wildlife might play a role in transmitting this pathogen to domestic animals.

*Ehrlichia* spp. Omatjenne has previously been reported in cattle and in ixodid ticks feeding on cattle in Turkey [25, 26]. However, none of the samples analysed in the present study were found to be positive for this pathogen.

### The rate of mixed infections and risk factors

The results of the present study illustrate that mixed infection of tick-borne pathogens are very common in small ruminants in Turkey. Risk factors that may influence the prevalence of TBHDs include age and immune status of the host population [64–66] and the distribution and seasonal activity of ticks [65, 66]. Potential vectors of these TBHDs have been reported in different parts of Turkey in the Black Sea, Mediterranean, southeastern, central Anatolia, eastern and Aegean regions [38, 44]. Our results highlight that although infections with *T. ovis* and/or *A. ovis* appear to be the main risk for small ruminants, there is also a clear risk of co-infection with species including *T. luwenshuni*, *T. uilenbergi*, *B. ovis*, *B. crassa*, *Babesia* spp. and *A. phagocytophilum*.

Host species susceptibility to tick-borne pathogens also plays a role in determining prevalence of disease. Goats are known to be more resistant to *T. lestoquardi* than sheep, however there is no evidence to suggest that sheep and goats differ in their susceptibility or resistance to other tick-borne pathogen species [2]. The results of the present study illustrate that sheep harbour significantly higher (*P* < 0.0001) numbers of single and/or mixed infections than goats (Table 4). At a species level, significantly higher (*P* < 0.0001) numbers of *T. ovis*, *T. luwenshuni*, *T. uilenbergi* and *Babesia* spp. infections were detected in sheep. In contrast to these, *B. motasi* and *A. phagocytophilum* were only detected in goats.

**Table 4:** Numbers of single and mixed species infections detected by RLB and PCR (no. of positive samples/no. of collected)

Region (no. of sheep/goat samples)	Sheep (*n* = 1727)^b,a^				Goats (*n* = 252)^b,a^			
	Single infection		Mixed infection		Single infection		Mixed infection	
	Detected by RLB	Detected by PCR	Detected by RLB	Detected by PCR	Detected by RLB	Detected by PCR	Detected by RLB	Detected by PCR
Aegean (863/160)	311/863	226/863	353/863	511/863	61/160	95/160	0/160	24/160
Mediterranean (350/32)	129/350	99/350	125/350	171/350	21/32	22/32	1/32	6/32
Central Anatolia (332/12)	130/332	95/332	141/332	217/332	0/12	0/12	0/12	0/12
Eastern Anatolia (32/0)	11/32	3/32	16/32	28/32	0/0	0/0	0/0	0/0
Southeastern Anatolia (150/48)	75/150	57/150	58/150	72/150	36/48	34/48	7/48	2/48
Total	656/1727	480/1727	693/1727	1001/1727	118/252	151/252	8/252	32/252
Percentage	33.1	24.3	35	50.6	6	7.7	0.4	1.6

^a^Statistically significant difference (*P* < 0.0001) between sheep and goats in terms of single and mixed infections detected by RLB and PCR

^b^Total number of samples collected from each species

The presence of multiple pathogens within an individual host may affect the outcome of infection. Co-infection with either *Theileria ovis*, *A. ovis* and *B. ovis* that are known to be non-pathogenic, mild pathogenic and highly pathogenic, respectively, can dramatically change the prognosis and increase the risk of severe disease and mortality. Additionally, it is known that TBF causes immunosuppression and infected animals become susceptible to secondary bacterial infections [67] that may result in decreased productivity and, in some cases, death [9]. It has been suggested that the outcome of concurrent infection with *A. ovis* and *B. ovis* can be more severe than a single infection alone [29]. Thus, co-infection with *A. ovis*/*A. phagocytophilum* and *B. ovis* may be associated with a poor prognosis and economic loss. Testing for only the major pathogen species may therefore underestimate the overall risk to livestock. Thus, assays that provide more comprehensive results will provide a better assessment of the impact on livestock productivity of co-infecting pathogens.

### Comparison of PCR and RLB

In field conditions, a proportion of animals will exist in a carrier state, not showing overt signs of disease. Detection of carriers is important, as these animals play a critical role in epidemiology of TBHDs by acting as reservoirs of infection for naive ticks and through movement they may introduce disease to new regions. For epidemiological studies, the requirement for a method capable of simultaneously detecting and discriminating these pathogens and other piroplasms is clear [68]. Molecular techniques, such as individual PCR, designed to detect single species have become very popular over the last two decades. In contrast, the RLB assay has the capacity to simultaneously detect and discriminate multiple species within a sample [4, 30]. In the present study, the efficacy of RLB hybridisation and species-specific PCR was compared in terms of their ability to detect single and mixed infections. For the detection of *A. ovis*, *T. ovis*, *T. luwenshuni*, *T. uilenbergi*, *Theileria* sp. MK, *B. ovis* and *A. phagocytophilum*, agreement between two tests ranged from none to moderate (Table 2). For *A. ovis*, nearly half (45%) of PCR positives were found to be negative with RLB, however only 12.3% of the *T. ovis* PCR positives were found to be negative with RLB (Table 2). Similarly, in a previous study, *T. lestoquardi* and *B. ovis* were detected using species-specific PCR in samples that were negative by RLB [29]. Variables such as the amount of template DNA in carrier animals [69, 70], the presence of multiple parasites in one sample [19] as well as competition for a finite amount of reagents between primers affect the amount of each product generated during the PCR reaction [71, 72]. This may compromise the efficacy of the test to amplify all parasite genotypes resulting a reduction in sensitivity and thus an underestimation of herd infection rates in comparison to RLB [19, 30]. Thus, in the case of single PCR assays when applied to samples containing a single species of pathogen, a high proportion of particular template DNA together with a lack of competition among primers would be predicted to result in a more sensitive reaction and a higher yield of amplicon. This was supported by the results obtained in the present study in which a statistically higher (P < 0.0001, Table 3) number of mixed infections were detected by PCR (52.24%) than RLB (35.42%). Interestingly, all RLB-positive samples for *T. uilenbergi* were found to be negative with species-specific PCR (Table 2). This difference between the two tests was observed in samples having multiple species and may be attributed to a low parasitaemia [73].

## Conclusions

The results obtained in this study indicate that the prevalence and distribution of *Theileria*, *Babesia*, *Anaplasma* and *Ehrlichia* species in small ruminants are complex and mixed infections are common throughout Turkey. Also, it should be appreciated that the failure to detect particular species in some regions does not necessarily indicate the absence of these pathogens. In order to assess risk factors associated with TBHDs and to improve design of cost-effective control strategies, it is important to utilise accurate diagnostic tests in the field [66]. It can be concluded from these observations that RLB is capable of detecting most, but not all, of the mixed infections in some blood samples and that the diagnostic sensitivity of species-specific, single PCR was superior. Therefore, the use of species-specific single PCR is recommended to better estimate the prevalence in regions characterised by high transmission rates where a large proportion of animals are co-infected.

## Additional files

Additional file 1: Table S1. Primers used for species-specific single PCR and 18S/16S PCRs for RLB hybridisation. (DOCX 92 kb)

Additional file 2: Table S2. Sequences and specificity of oligonucleotide probes used for RLB hybridisation assay. (DOCX 111 kb)

Additional file 3: Table S3. Conditions for each species-specific single PCR. (DOCX 70 kb)

Additional file 4: Table S4. Mixed species infections detected by RLB. (DOCX 116 kb)

Additional file 5: Figure S1. Distribution of single, mixed and total infections detected by RLB in each region (a) and among 18 provinces (b). (DOCX 2154 kb)

Additional file 6: Table S5. Mixed species infections detected by species-specific single PCR. (DOCX 101 kb)

Additional file 7: Figure S2. Distribution of single, mixed and total infections detected by PCR in each region (a) and among 18 provinces (b). (DOCX 1785 kb)

## Abbreviations

RLB: Reverse line blotting
TBF: Tick-borne fever
TBHDs: Tick borne haemoparasitic diseases
