# Supplementary material for: Prevalence of tick-borne haemoparasites in small ruminants in Turkey and diagnostic sensitivity of single-PCR and RLB
Source: Parasit Vectors. 2017 Apr 27;10:211. doi: 10.1186/s13071-017-2151-3 (PMC5408456; doi:10.1186/s13071-017-2151-3)
Supplement: Supplementary file 1 — Primers used for species-specific single PCR and 18S/16S PCRs for RLB hybridisation. (DOCX 92 kb) [file 13071_2017_2151_MOESM1_ESM.docx]

**Table S1.** Primers used for species-spesific single PCR and 18S/16S PCRs for RLB hybridization

| **Target Gene** | **Primer_ID** | **Sequences^a^** | **Specificity** | **References** |
| --- | --- | --- | --- | --- |
| 18S ssu rRNA | RLB-F/  RLB-R | F; GACACAGGGAGGTAGTGACAAG  R; 5’-biotin-CTAAGAATTTCACCTCTGACAGT | All *Theileria* and *Babesia* species | [30] |
| 16S ssu rRNA | Ehr-F/  Ehr-R | F; GGAATTCAGAGTTGGATCMTGGYTCAGR;  5’-biotin- CGGGATCCCGAGTTTGCCGGGACTTYTTCT | All *Ehrlichia* and *Anaplasma* species | [8] |
| 18S ssu rRNA | TSsr 170F/  TSsr 670R | F; TCGAGACCTTCGGGT  R; TCCGGACATTGTAAAACAAA | *T.ovis* | [37] |
| Merozoite surface antigen | TlestF/  TlestR | F; GTGCCGCAAGTGAGTCA  R; GGACTGATGAGAAGACGATGAG | *T.lestoquardi* | [14] |
| 18S ssu rRNA | Bov-F/  Bov-R | F; TGGGCAGGACCTTGGTTCTTCT  R; CCGCGTAGCGCCGGCTAAATA | *B.ovis* | [39] |
| 18S ssu rRNA | Tuil310s/  Tuil 689as | F; GGTAGGGTATTGGCCTACCGG  R; ACACTCGGAAAATGCAAGCA | *T. uilenbergi* | [6] |
| 18S ssu rRNA | Tluw310s/  Tluw 374as | F; GGTAGGGTATTGGCCTACTGA  R;TCATCCGGATAATACAAGT | *T. luwenshuni* | [6] |
| 18S ssu rRNA | Tmk-F/990 | F; CATTGTTTCTTCTCATGTC  R; TTGCCTTAAACTTCCTTG | *T.sp* MK | [39] |
| Major surface antigen (*msp*) | MSP4-F/  MSP4-R | F; ATGAATTACAGAGAATTGCTTGTAGG  R; TTAATTGAAAGCAAATCTTGCTCCTATG | *A.phagocytophilum* | [55] |
| Major surface antigen (*msp*) | MSP45-F/  MSP45-R | F; GGGAGCTCCTATGAATTACAGAGAATTGTTTAC  R; CCGGATCCTTAGCTGAACAGGAATCTTGC | *A. ovis* | [53] |

(^a^); Primer sequences are given in 5'-3' direction.

(^b^); ‘F’ and ‘R’ indicates forward and reverse primers, respsctively.

(^c^); Degenerate primers with ‘R’ indicates A or G bases, ‘Y’ indicates C or T bases, ‘M’ indicates A or C bases, ‘W’ indicates A or T bases and ‘K’ indicates G or T bases in that position.
