# Supplementary material for: Prevalence of tick-borne haemoparasites in small ruminants in Turkey and diagnostic sensitivity of single-PCR and RLB
Source: Parasit Vectors. 2017 Apr 27;10:211. doi: 10.1186/s13071-017-2151-3 (PMC5408456; doi:10.1186/s13071-017-2151-3)
Supplement: Supplementary file 2 — Sequences and specificity of oligonucleotide probes used for RLB hybridisation assay. (DOCX 111 kb) [file 13071_2017_2151_MOESM2_ESM.docx]

**Table S2.** Sequences and specificity of oligonucleotide probes used for RLB hibridisation assay

| **Prob names*** | **Oligonucleotide prob sequences^a^** | **Specificity** | **References** |
| --- | --- | --- | --- |
| *T/B* catchall^c^ | TAATGGTTAATAGGARCRGTTG | All *Theileria* and *Babesia* spp. | [4] |
| *Theileria* all | TGATGGGAATTTAAACCYCTTCCA | All *Theileria spp.* | [4] |
| *T ovis* | TTGCTTTTGCTCCTTTACGAGTCTTTGC | *T.ovis* | [4] |
| *T lesto* | CTTGTGTCCCTCCGGG | *T.lestoquardi* | [4] |
| *T lesto*II | ATTGCTTGTGTCCCTCCG | *T.lestoquardi* | [4] |
| *T uilenbergi* | TGCATTTTCCGAGTGTTACT | *T.uilenbergi* | [4] |
| *T luwenshuni* | TCGGATGATACTTGTATTATC | *T.luwenshuni* | [4] |
| T sp.OT1 | ATCTTCTTTTTGATGAGTTGGTGT | *T.spp* OT1 | [11] |
| T sp. OT3 | ATTTTCTCTTTTTATATGAGTTTT | *T.spp* OT3 | [11] |
| T sp. MK | CATTGTTTCTTCTCATGTC | *T.sp* MK | [11] |
| *T separata* | GGTCGTGGTTTTCCTCGT | *T.seperata* | [4] |
| B all | CCTKGGTAATGGTTAATAGGAA | All *Babesia spp.* | [4] |
| *B ovis* | TGCGCGCGGCCTTTGCGTTACT | *B.ovis* | [4, 11] |
| Bm3** | TTTCAAGCAGACTTTTGTCTTG | *B. motasi, B. sp.* China, *B. crassa* spp. | [4] |
| Bm2-2** | GAATGATGCCGACTTAAACCCT | *B. motasi, B.sp.* China spp. | [4] |
| Bm1** | GCTTGCTTTTTTGTTACTTTTG | *B.motasi* | [4] |
| *B.motasi* | ATTGGAGTATTGCGCTTGCTTTTT | *B.motasi* | [11] |
| BcG*** | GTTGGCTTATCTTTTTACTTT | *B. crassa* group | [4] |
| Bcİ*** | TTATGGCCCGTTGGCTTAT | *B. crassa* Iran | [4] |
| BcT*** | tctgatcgagttggctta | *B. crassa* Turkey | [4] |
| E/A catch-all | GGGGGAAAGATTTATCGCTA | All *Ehrlichia* and *Anaplasma* spp. | [8] |
| *A ovis* | ACCGTACGCGCAGCTTG | *A.ovis* | [8] |
| *A phago* I**** | TTGCTATAAAGAATAATTAGTGG | *A.phagocytophilum* | [8] |
| *A phago* II**** | TTGCTATGAAGAATAATTAGTGG | *A.phagocytophilum* | [8] |
| *A phago* III**** | TTGCTATAAAGAATAGTTAGTGG | *A.phagocytophilum* | [8] |
| *A phago* IV**** | TTGCTATAGAGAATAGTTAGTGG | *A.phagocytophilum* | [8] |
| *E rum* | AGTATCTGTTAGTGGCAG | *E.ruminantium* | [8] |
| *E ovina* | TCTGGCTATAGGAAATTGTTA | *E.ovina* | [8] |
| *E spp* Omatjane | CGGATTTTTATCATAGCTTGC | *E.spp.* Omatjane | [8] |

(^a^); Oligonucleotide probes with ‘R’ indicates A or G bases, ‘Y’ indicates C or T bases, ‘M’ indicates A or C bases, ‘W’ indicates A or T bases and ‘K’ indicates G or T bases in that position.

(^*^); Sequences are given in 5'-3' direction. (**); Bm3 indicates; *Babesia motasi, Babesia sp*. China, *Babesia crassa*, Bm2-2 indicates; *Babesia motasi*, *Babesia sp.* China and Bm1 indicates; *Babesia motasi*. (***); BcG indicates;*Babesia crassa* group; B cI indicates; *Babesia crassa* Iran and BcT indicates; *Babesia crassa* Turkey. (****); indicates different isolates of *A.phagocytophilum*
