# Supplementary material for: Prevalence of tick-borne haemoparasites in small ruminants in Turkey and diagnostic sensitivity of single-PCR and RLB
Source: Parasit Vectors. 2017 Apr 27;10:211. doi: 10.1186/s13071-017-2151-3 (PMC5408456; doi:10.1186/s13071-017-2151-3)
Supplement: Supplementary file 3 — Conditions for each species-specific single PCR. (DOCX 70 kb) [file 13071_2017_2151_MOESM3_ESM.docx]

**Table S3.** Conditions for each species-spesific single PCR

| **Species** | **Amplicon size (bp)** | **Initial Denaturation** | **Number of Cycles** x [Denaturation-Primer Annealing- Extension**]** | **Final Extension** |
| --- | --- | --- | --- | --- |
| *A.ovis* | 870 | 1x 95°C 12m | 35x [95°C 50sec-60°C 50sec-65°C 1m] | 1x 65°C 10m |
| *A.phagocytophilum* | 849 | 1x 95°C 12m | 35x [95°C 50sec-61°C 50sec-72°C 30sec] | 1x 72°C 10m |
| *B.ovis* | 549 | 1x 95°C 12m | 35x [95°C 50sec-60°C 50sec-72°C 50sec] | 1x 72°C 10m |
| *T. lestoquardi* | 730 | 1x 95°C 12m | 35x [95°C 50sec-54°C 50sec-72°C 50sec] | 1x 72°C 10m |
| *T. luwenshuni* | 388 | 1x 95°C 12m | 35x [95°C 50sec-57°C 50sec-72°C 50sec] | 1x 72°C 10m |
| *T.uilenbergi* | 389 | 1x 95°C 12m | 35x [95°C 50sec-55°C 50sec-72°C 50sec] | 1x 72°C 10m |
| *T.ovis* | 520 | 1x 95°C 12m | 35x [95°C 50sec-55°C 50sec-72°C 50sec] | 1x 72°C 10m |
| *T. sp. MK* | 757 | 1x 95°C 12m | 35x [95°C 50sec-60°C 50sec-65°C 1m] | 1x 65°C 10m |
