# Supplementary material for: Prevalence of tick-borne haemoparasites in small ruminants in Turkey and diagnostic sensitivity of single-PCR and RLB
Source: Parasit Vectors. 2017 Apr 27;10:211. doi: 10.1186/s13071-017-2151-3 (PMC5408456; doi:10.1186/s13071-017-2151-3)
Supplement: Supplementary file 4 — Mixed species infections detected by RLB. (DOCX 116 kb) [file 13071_2017_2151_MOESM4_ESM.docx]

**Table S4.** Mixed species infections detected by RLB

| **Province** | **No. of animals** | **RLB* (sheep/goat)** | | | | | | | | | | | | | | | | | | | | | | | | | | | | | | | | |
| --- | --- | --- | --- | --- | --- | --- | --- | --- | --- | --- | --- | --- | --- | --- | --- | --- | --- | --- | --- | --- | --- | --- | --- | --- | --- | --- | --- | --- | --- | --- | --- | --- | --- | --- |
|  |  | ***A.o/B.o*** | ***A.o/T.o*** | ***A.o/B.sp*** | ***A.o/Bc*** | ***A.o/T.u*** | ***A.o/T.OT1*** | ***A.o/T.OT3*** | ***B.o/T.o*** | ***T.o/B.sp*** | ***T.o/Bc*** | ***T.o/T.MK*** | ***T.o/T.OT1*** | ***T.u/Bc*** | ***T.OT1/Bc*** | ***T.OT1/T.u*** | ***A.o/B.o/T.o*** | ***A.o/T.o/Bsp*** | ***A.o/T.o/T.MK*** | ***A.o/T.o/Bc*** | ***A.o/T.o/T.OT1*** | ***A.o/T.OT1/Bsp*** | ***A.o/T.OT1/Bc*** | ***A.o/T.OT1/T.l*** | ***T.o/T.OT1/Bc*** | ***T.o/T.MK/Bc*** | ***T.o/T.MK/Bsp*** | ***T.o/T.OT3/Bsp*** | ***Ao/To/Tu/Bc*** | ***Ao/To/Tu/TOT1*** | ***Ao/To/TOT1/Bc*** | ***Ao/Bo/To/T.sep*** | ***To/Tu/TOT1/Bc*** | ***A.o/T.o/T.u/T.OT1/Bc*** |
|  |  |  |  |  |  |  |  |  |  |  |  |  |  |  |  |  |  |  |  |  |  |  |  |  |  |  |  |  |  |  |  |  |  |  |
| **Adana** | 95 |  | 48 |  |  | 1^a^ |  |  |  |  |  |  |  |  |  | 1 |  |  | 1 |  |  |  |  |  |  |  |  |  |  |  |  |  |  |  |
| **Afyon** | 100 |  | 23 |  |  |  |  |  |  | 15 |  |  |  |  |  |  |  |  |  |  |  |  |  |  |  |  | 1 | 2 |  |  |  |  |  |  |
| **Aksaray** | 55 |  | 20 |  | 1 |  |  |  |  |  |  |  | 1 |  |  |  |  |  |  | 1 | 1 | 1 |  |  | 1 |  |  |  |  |  |  |  |  |  |
| **Antalya** | 95 |  |  |  |  |  |  |  |  |  |  |  |  |  |  |  |  |  |  |  |  |  |  |  |  |  |  |  |  |  |  |  |  |  |
| **Aydın** | 273 |  | 102 |  |  |  |  |  |  | 1 |  |  |  |  |  |  |  |  |  |  |  |  |  |  |  |  |  |  |  |  |  |  |  |  |
| **Burdur** | 137 |  | 38 |  |  |  |  |  |  | 3 | 1 |  | 1 |  | 2 |  |  | 5 |  | 1 |  |  |  |  |  |  |  |  |  |  |  |  |  |  |
| **Denizli** | 140 |  | 50 |  |  |  |  |  |  |  | 6 | 2 | 4 |  | 1 |  |  |  |  | 9 |  |  |  |  | 3 | 1 |  |  | 1 | 1 | 3 |  |  |  |
| **Isparta** | 55 |  | 4 |  |  |  |  |  |  | 12 |  |  |  |  |  |  |  | 8 |  |  |  |  |  |  |  |  |  |  |  |  |  |  |  |  |
| **İzmir** | 104 |  | 14 |  |  |  |  |  | 1 |  |  |  |  |  |  |  |  |  |  |  |  |  |  |  |  |  |  |  |  |  |  |  |  |  |
| **Konya** | 75 |  | 18 |  |  |  |  |  |  | 8 | 3 |  |  |  |  |  |  | 8 |  | 2 |  |  |  |  |  |  |  |  |  |  |  |  |  |  |
| **Kütahya** | 103 | 2 | 17 | 3 |  |  |  |  |  | 10 |  |  |  |  |  |  |  | 19 |  |  |  |  |  |  |  |  |  |  |  |  |  |  |  |  |
| **Manisa** | 101 |  | 6 |  |  |  |  |  |  | 6 |  |  | 1 |  |  |  |  |  |  |  | 1 |  |  |  |  |  |  |  |  |  |  |  |  |  |
| **Muğla** | 114 |  |  |  |  |  |  |  |  |  |  |  |  |  |  |  |  |  |  |  |  |  |  |  |  |  |  |  |  |  |  |  |  |  |
| **Niğde** | 214 | 2 | 59 |  | 4 |  | 4 |  |  | 1 |  |  | 1 |  |  |  |  |  |  |  | 2 |  |  | 1 |  |  |  |  |  | 1 |  | 1 |  |  |
| **Şırnak** | 98 |  | 27/4 |  | 1^a^ |  | 1^a^ | 1^a^ |  |  |  |  |  |  |  |  | 1 |  | 1 |  |  |  |  |  |  |  |  |  |  |  |  |  |  |  |
| **Şanlıurfa** | 100 |  | 7 |  |  |  |  |  |  |  | 9 |  | 2 | 3 | 2 |  |  |  |  | 1 |  |  | 1 |  | 2 |  |  |  |  |  |  |  | 1 | 1 |
| **Uşak** | 88 |  | 41 |  | 1 |  |  |  |  |  | 1 |  |  |  | 2 |  |  |  |  | 1 | 1 |  | 1 |  |  |  |  |  |  |  |  |  |  |  |
| **Van** | 32 |  | 15 |  |  |  |  |  |  |  |  |  |  |  |  |  |  |  | 1 |  |  |  |  |  |  |  |  |  |  |  |  |  |  |  |
| **TOTAL** | **1979** | **4** | **493** | **3** | **7** | **1** | **5** | **1** | **1** | **56** | **20** | **2** | **10** | **3** | **7** | **1** | **1** | **40** | **3** | **15** | **5** | **1** | **2** | **1** | **6** | **1** | **1** | **2** | **1** | **2** | **3** | **1** | **1** | **1** |
| **Percentage** | | **0.2** | **24.8** | **0.2** | **0.4** | **0.1** | **0.3** |  |  | **2.8** | **1.0** | **0.1** | **0.5** | **0.2** | **0.4** |  |  | **2.0** | **0.2** | **0.8** | **0.3** |  | **0.1** |  | **0.3** |  |  | **0.1** |  | **0.1** | **0.2** |  |  |  |

***A.o=****Anaplasma ovis,* ***B.o=****Babesia ovis,* ***T.o=****Theileria ovis,* ***T.l=****Theileria luwenshuni,* ***T.u=****Theileria uilenbergi,* ***T.OT1=****Theileria* sp. OT1*,* ***T.OT3=****Theileria* sp. OT3*,* ***T.MK=****Theileria* sp. MK*,* ***Tall=*** indicates samples that showed reactivity with *Theileria* all probe and considered as *Theileria* spp positive, ***Ball=*** indicates samples that showed reactivity with *Babesia* all probe and considered as *Babesia* spp positive*,* ***BcG*=** *Babesia crassa* group*,* ***T.sep***=*Theileria separata*. **^a^** number of positive samples collected from goats.
