# Supplementary material for: Prevalence of tick-borne haemoparasites in small ruminants in Turkey and diagnostic sensitivity of single-PCR and RLB
Source: Parasit Vectors. 2017 Apr 27;10:211. doi: 10.1186/s13071-017-2151-3 (PMC5408456; doi:10.1186/s13071-017-2151-3)
Supplement: Supplementary file 5 — Distribution of single, mixed and total infections detected by RLB in each region (a) and among 18 provinces (b). (DOCX 2154 kb) [file 13071_2017_2151_MOESM5_ESM.docx]

**
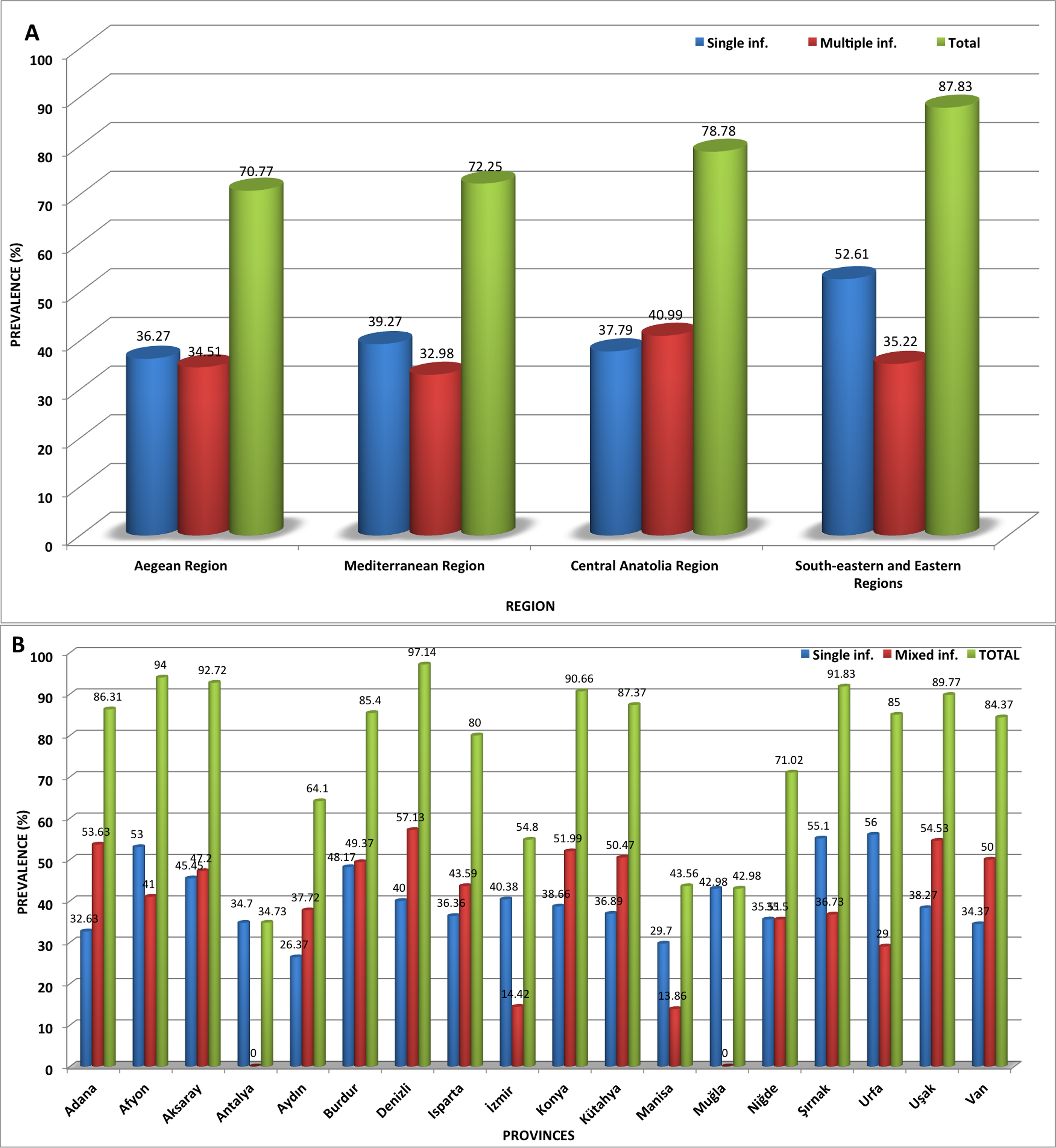
**

**Figure S1.** Distibution of single, mixed and total infections detected by RLB in each region (**a**) and among 18 different provinces (**b**).
