# Supplementary material for: Prevalence of tick-borne haemoparasites in small ruminants in Turkey and diagnostic sensitivity of single-PCR and RLB
Source: Parasit Vectors. 2017 Apr 27;10:211. doi: 10.1186/s13071-017-2151-3 (PMC5408456; doi:10.1186/s13071-017-2151-3)
Supplement: Supplementary file 6 — Mixed species infections detected by species-specific single PCR. (DOCX 101 kb) [file 13071_2017_2151_MOESM6_ESM.docx]

**Table S5.** Mixed species infections detected by species-specific single PCR

| **Provinces** | **No. of animals** |  | **PCR (sheep/goat)** | | | | | | | | | | | | | | | | | | | | | | | | | | |
| --- | --- | --- | --- | --- | --- | --- | --- | --- | --- | --- | --- | --- | --- | --- | --- | --- | --- | --- | --- | --- | --- | --- | --- | --- | --- | --- | --- | --- | --- |
|  |  | ***Ao/Bo*** | ***Ao/To*** | ***Ao/TI*** | ***Ao/Tu*** | ***Ao/TMK*** | ***Ao/Ap*** | ***To/Bo*** | ***TI/Bo*** | ***To/TI*** | ***To/Tu*** | ***To/TMK*** | ***Tl/TMK*** | ***Ao/To/Bo*** | ***Ao/Bo/Tu*** | ***Ao/Bo/Ap*** | ***Ao/To/Tl*** | ***Ao/To/Tu*** | ***Ao/To/TMK*** | ***Ao/Tl/Tu*** | ***To/Tl/Bo*** | ***To/Tu/Bo*** | ***To/Tl/Tu*** | ***To/Tu/TMK*** | ***Ao/To/Bo/Tl*** | ***Ao/To/Bo/Tu*** | ***Ao/To/Tl/Tu*** | ***Ao/To/Tl/TMK*** | ***Ao/To/Tu/TMK*** |
| **Adana** | **95** |  | 41/1 |  |  |  | 1^a^ |  |  |  |  |  |  |  |  |  |  |  | 1 |  |  |  |  |  |  |  |  |  |  |
| **Afyon** | **100** | 1 | 57 |  |  |  |  | 7 |  |  |  |  |  | 17 |  |  |  | 2 | 2 |  |  |  |  |  |  | 1 |  |  |  |
| **Aksaray** | **55** |  | 22 |  |  |  |  | 2 |  |  |  |  |  | 9 |  |  |  |  |  |  |  |  |  |  |  |  |  |  |  |
| **Antalya** | **95** |  | 1 |  | 2 |  |  |  |  |  | 2 |  |  |  |  |  |  | 1 |  |  |  |  |  |  |  |  |  |  |  |
| **Aydın** | **273** | 1 | 63 | 12 | 1 | 1 |  | 1 | 1 | 1 | 6 | 1 |  |  |  |  |  | 4 | 1 |  |  |  |  |  |  |  |  |  |  |
| **Burdur** | **137** | 1^a^ | 67/1 |  | 3/1 |  |  |  |  |  | 2 |  |  | 2 | 1 |  | 1 | 3/1 | 1 |  |  |  |  |  |  |  | 1 |  |  |
| **Denizli** | **140** |  | 64 |  |  |  |  |  |  | 4 | 4 |  |  |  |  |  | 6 | 24 | 1 | 1 |  |  |  | 1 |  |  | 6 | 1 |  |
| **Isparta** | **55** |  | 39 |  |  |  |  |  |  | 3 |  |  |  |  |  |  |  |  |  |  |  |  |  |  |  |  |  |  |  |
| **İzmir** | **104** |  | 31 |  |  | 2 |  |  |  | 1 |  | 4 | 1 |  |  |  | 3 | 3 | 11 |  |  |  |  |  |  |  |  | 4 |  |
| **Konya** | **75** |  | 48 |  |  |  |  |  |  |  |  |  |  | 8 |  |  |  |  | 1 |  |  |  |  |  |  |  |  |  |  |
| **Kütahya** | **103** | 9 | 32 |  | 1 |  |  |  |  |  |  | 1 |  | 13 | 1 |  |  | 1 |  |  |  |  |  |  |  |  |  |  |  |
| **Manisa** | **101** |  | 33 |  |  |  |  |  |  |  |  |  |  | 3 | 1 |  |  | 5 |  |  |  |  |  |  |  | 1 |  |  |  |
| **Muğla** | **114** | 1^a^ |  |  |  |  | 22^a^ |  |  |  |  |  |  |  |  | 1^a^ |  |  |  |  |  |  |  |  |  |  |  |  |  |
| **Niğde** | **214** |  | 56 | 13 |  |  |  |  | 2 | 16 |  |  |  | 2 |  |  | 31 |  |  |  | 2 |  |  |  | 5 |  |  |  |  |
| **Şırnak** | **98** | 1^a^ | 29/1 |  |  |  |  |  |  |  |  |  |  | 1 |  |  |  |  | 2 |  |  |  |  |  |  |  |  |  |  |
| **Şanlıurfa** | **100** |  | 27 |  |  |  |  |  |  | 6 | 2 |  |  |  |  |  | 1 | 1 | 3 |  |  |  |  |  |  |  |  |  |  |
| **Uşak** | **88** |  | 45 |  |  |  |  |  |  |  | 2 | 2 |  |  |  |  |  | 7 | 4 |  |  |  |  |  |  |  |  |  |  |
| **Van** | **32** |  | 5 |  | 6 |  |  |  |  |  | 1 |  |  |  |  |  |  | 12 |  | 1 |  |  | 1 |  |  |  |  |  | 2 |
| **TOTAL** | **1979** | **14** | **663** | **25** | **14** | **3** | **23** | **10** | **3** | **31** | **19** | **8** | **1** | **55** | **3** | **1** | **42** | **64** | **27** | **2** | **2** | **1** | **1** | **1** | **5** | **2** | **7** | **5** | **2** |
| **Percentage,%** | | **0.7** | **33.5** | **1.3** | **0.7** | **0.2** | **1.2** | **0.5** | **0.2** | **1.6** | **1** | **0.4** |  | **2.8** | **0.2** |  | **2.1** | **3.2** | **1.4** | **0.1** | **0.1** |  |  |  | **0.3** | **0.1** | **0.4** | **0.3** | **0.1** |

***A.o=****Anaplasma ovis,* ***B.o=****Babesia ovis,* ***T.o=****Theileria ovis,* ***T.l=****Theileria luwenshuni,* ***T.u=****Theileria uilenbergi,* ***T.le=****Theileria lestoquardi,* ***T.MK=****Theileria* sp. MK, ***A.p=****Anaplasma phagocytophilum*. **^a^** number of positive samples collected from goats.
